# Laser capture microdissection of intestinal tissue from sea bass larvae using an optimized RNA integrity assay and validated reference genes

**DOI:** 10.1038/srep21092

**Published:** 2016-02-17

**Authors:** M. Schaeck, W. De Spiegelaere, J. De Craene, W. Van den Broeck, B. De Spiegeleer, C. Burvenich, F. Haesebrouck, A. Decostere

**Affiliations:** 1 Department of Morphology, Faculty of Veterinary Medicine, Ghent University, Salisburylaan 133, 9820 Merelbeke, Belgium; 2 Department of Internal Medicine, Faculty of Medicine and Health Sciences, Ghent University, De Pintelaan 185, 9000 Ghent, Belgium; 3 Department of Pharmaceutical Analysis, Laboratory of Drug Quality & Registration, Faculty of Pharmaceutical Sciences, Ghent University, Ottergemsesteenweg 460, 9000 Ghent, Belgium; 4 Department of Comparative Physiology and Biometrics, Faculty of Veterinary Medicine, Ghent University, Salisburylaan 133, 9820 Merelbeke, Belgium; 5 Department of Pathology, Bacteriology and Avian Diseases, Ghent University, Salisburylaan 133, 9820 Merelbeke, Belgium

## Abstract

The increasing demand for a sustainable larviculture has promoted research regarding environmental parameters, diseases and nutrition, intersecting at the mucosal surface of the gastrointestinal tract of fish larvae. The combination of laser capture microdissection (LCM) and gene expression experiments allows cell specific expression profiling. This study aimed at optimizing an LCM protocol for intestinal tissue of sea bass larvae. Furthermore, a 3′/5′ integrity assay was developed for LCM samples of fish tissue, comprising low RNA concentrations. Furthermore, reliable reference genes for performing qPCR in larval sea bass gene expression studies were identified, as data normalization is critical in gene expression experiments using RT-qPCR. We demonstrate that a careful optimization of the LCM procedure allows recovery of high quality mRNA from defined cell populations in complex intestinal tissues. According to the geNorm and Normfinder algorithms, *ef1a, rpl13a, rps18* and *faua* were the most stable genes to be implemented as reference genes for an appropriate normalization of intestinal tissue from sea bass across a range of experimental settings. The methodology developed here, offers a rapid and valuable approach to characterize cells/tissues in the intestinal tissue of fish larvae and their changes following pathogen exposure, nutritional/environmental changes, probiotic supplementation or a combination thereof.

The increasing demand for a sustainable aquaculture industry has promoted research regarding optimal environmental and nutritional parameters so as to prevent diseases^1^. Nevertheless, the larval phase of the aquaculture production cycle remains affected by a high susceptibility to diseases, thereby engendering a major bottleneck for the sustainable expansion of aquaculture. The mucosal surface of the intestinal tract is known to constitute the first line of defense against pathogen invasion^2^. Therefore, many areas of intense research have hinged upon a better understanding of intestinal mucosal health, with a focus on the impact of nutrition, immunostimulants, pre- and probiotics, and exposure to pathogens^2^.

Transcriptomic methods offer a rapid and valuable approach to characterize the aforementioned impacts on intestinal mucosal health^3^. However, studies resorting to gene expression analyses in fish larvae remain scarce, and most of these studies employ homogenized whole larval bodies or segments for RNA extraction. This is rooted in the small size of fish larvae in the first weeks following hatching, which does not allow to specifically isolate the intestinal tract with conventional dissection techniques. In this way, interesting phenomena occurring at the intestinal level may be concealed by dissimilar gene expression levels in the various organs and tissues^4^,^5^,^6^.

Laser capture microdissection (LCM)^7^ circumvents the difficult sampling of the tiny fish larvae by allowing researchers to isolate specific cell populations or individual cells from sections of complex tissues^8^. LCM coupled with reverse transcription quantitative polymerase chain reaction (RT-qPCR) indeed is a powerful method to accurately determine gene expression in particular cell types^9^, rendering it an ideal tool to quantify gene regulatory effects and elucidate the molecular basis of functional feeds or pathogenesis^10^,^11^. To date, only one study presented a protocol for successful LCM in fish larvae, isolating the gut contents from larval cod (*Gadus morhua*) for a more accurate diet analysis^12^. LCM followed by RT-qPCR to assess gene expression in intestinal tissue has not been optimized yet in fish larvae. When doing so, various success-determining challenges need to be addressed. The time consuming process of LCM may elicit ribonucleic acid (RNA) degradation, which may extend dramatically depending upon manipulations and tissue type. Notably, intestinal tissues are known to maintain the poorest RNA integrity with fast degradation, hampering the downstream applications such as gene expression analysis^13^. Consequently, RNA integrity needs to be assessed to enable a proper evaluation of the biological relevance of RT-qPCR data.

In addition to this, the choice of a proper normalization method to correct for sample input is crucial^14^,^15^. The exponential amplification, which makes RT-qPCR an extremely powerful method to quantify low levels of template DNA molecules in a sample, also increases variation, as minor technical variations in sample input are being magnified. It is now generally agreed that the use of multiple internal reference genes, which were previously validated to be stably expressed in the specific experimental conditions, offers the most optimal method for RT-qPCR normalization^16^,^17^.

In the current study, we strived to optimize an LCM protocol to procure intestinal tissue from fish larvae whilst maintaining a high RNA quality for downstream gene expression analysis by RT-qPCR. European sea bass larvae (*Dicentrarchus labrax*, Linnaeus, 1758) was employed as a model species, as it is the most extensively cultured seawater fish species from the Mediterranean. For that purpose, fixation protocols were compared focusing on preserving tissue morphology and RNA integrity, two critical success factors for LCM and consequent RT-qPCR. Furthermore, the most suitable reference genes, stably expressed in specific experimental conditions, for performing RT-qPCR in the intestinal tract of larval sea bass gene expression studies were determined. Therefore, four experimental groups, covering three different microbiological conditions and two developmental stages, were included in this study i.e. conventional larvae (CON) of 16 days post hatching (dph); germ-free larvae (GF) of 10 dph; GF larvae of 16 dph; and germ-free larvae supplemented with a probiotic candidate (GFPr) of 10 dph.

## Results

### Optimization of the fixation protocol

Methacarn fixation and snap freezing were evaluated on 10 CON larvae of 16 dph with regard to the maintenance of tissue morphology and RNA quality. Both fixation methods rendered satisfactory results in terms of preserving the morphology and staining by haematoxylin and eosin. Methacarn fixation consistently resulted in a superior morphology with an average quality score of 2.0 on a scale of 3 (i.e. fair quality), as assessed by two histologists. Snap frozen tissue rendered a mean score of 1.5 but no difficulties were encountered in clearly distinguishing morphological features of the intestinal tissue.

To assess RNA integrity, RNA from tissue scrapes were analyzed by automated electrophoresis using the Experion system. The best results were depicted for the snap frozen tissue sections with RNA quality indicator (RQI) values ranging from 7.0 to 9.1. Methacarn-fixed tissue showed a high RNA degradation with RQI values ranging from 1.9 to 2.4.

### RNA integrity assessment of LCM samples

As the fixation protocol based on snap freezing gave the best results regarding the maintenance of adequate morphology and RNA integrity, this protocol was adopted to fixate the 40 larvae, including 10 larvae from each experimental group i.e. CON 16 dph, GF 10 dph, GF 16 dph and GFPr 10 dph. Following fixation, intestinal tissue was microdissected and RNA integrity was assessed using both the Experion automated electrophoresis system and a newly developed 3′:5′ RT-qPCR assay for sea bass.

The Experion automated electrophoresis system gave a reliable RQI for merely seven out of 40 LCM samples, with a mean value of 7.7 ± 0.7 SD indicating good quality. Indeed, for the vast majority of samples, the RNA quantity with a mean of 179 pg ± 114 SD fell below the lower limit of 200 pg, that is considered the minimal RNA quantity to make an accurate assessment of its integrity according to the Manufacturers’ guidelines of the Experion system. This threshold was manually overruled and the (unreliable) values are displayed between brackets (Table 1).

**Figure 1:**
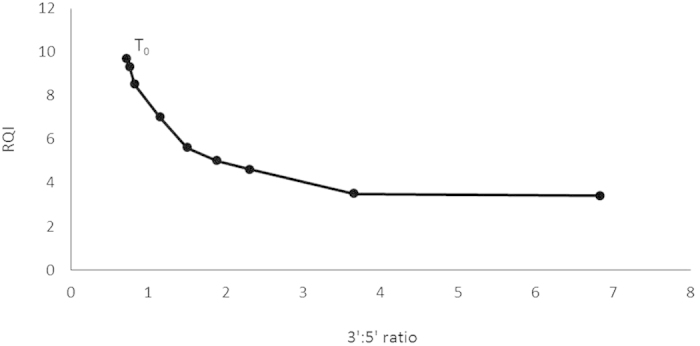
Degradation curve correlating RQI with 3′:5′ratio. Degradation was induced by exposure to 80 °C and every ten minutes a sample was processed and this for nine time points. T_0_ represents non-heat degraded RNA.

As automated electrophoresis is not able to provide reliable RNA integrity measurements for all LCM samples, we optimized the 3′:5′ RT-qPCR as a more sensitive assay to assess RNA integrity of intestinal tissue LCM samples of sea bass larvae. Both primer pairs amplified a single PCR product and showed an average efficiency of 99% (3′) and 100% (5′). For the 40 LCM samples, 3′:5′ ratios ranged from 0.5 to 2.4 (Table 1). To allow a reliable correlation between 3′:5′ ratios and RQI values a degradation curve of RNA by heat degradation was generated from a whole-body RNA sample from a CON 16 dph larvae (Fig. 1). Using this degradation curve, 3′:5′ ratios of all LCM samples could be reliably correlated with RQI values. According to this, only four out of forty samples reported an interpolated RQI value below 5.

### Reference gene evaluation

**Figure 2:**
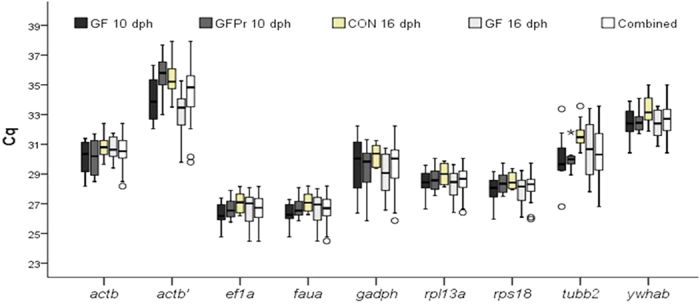
Expression levels of candidate reference genes (Cq values). of the four larval groups separately and in combination. Bars indicate the 25/75 percentiles, whisker caps indicate the 10/90 percentile, the horizontal line marks the median and all outliers are indicated by dots. CON: conventional larvae; GF: germ-free larvae and GFPr: germ-free larvae supplemented with a probiotic candidate.

To evaluate the stability of candidate reference genes, the expression levels of the reference genes were measured over the 40 RNA samples originating from the four experimental groups, including 10 larval samples per group (Fig. 2). The smallest Cq variance was exhibited by *rps18* (0.65) *rpl13a* (0.70), *ef1a* (0.72) and *faua* (0.70). In contrast, *actb*′ (3.23), *tubb2* (2.36) and *gapdh* (2.41) displayed a high expression variance in sea bass larvae.

The mRNA expression profiles for all reference genes studied for all experimental conditions showed little variation between the groups (Fig. 2). The most pronounced variation between groups was found in the expression level of *actb*′. Reference genes *ef1a* and *faua* showed the highest expression in the four groups, whereas *actb*′ and *ywhab* showed the lowest expression.

**Figure 3:**
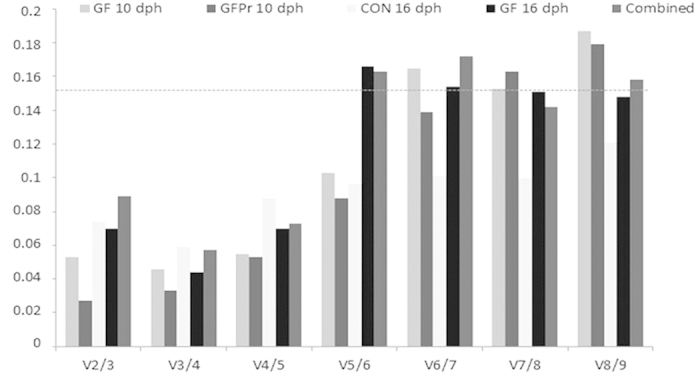
Determination of the optimal number of reference genes. Pairwise variation calculated by geNorm to determine the minimum number of reference genes for accurate normalization in all the samples (Combined), or in the samples of the experimental condition of interest. Every bar represents change in normalization accuracy when stepwise adding more endogenous reference genes according to the ranking in Fig. 3. CON: conventional larvae; GF: germ-free larvae and GFPr: germ-free larvae supplemented with a probiotic candidate.

The gene expression stability analysis over the individual subgroups by geNorm ranked the genes based on their stability measure value. For all subgroups, ef1a, faua, rps18 and rpl13a were the most stable genes with M-values not exceeding 0.3. Generally, the differences regarding age or treatment between the subgroups had little effect on the order of stability for the majority of the genes (Table 2). Additionally, a combined analysis was performed to determine the most suitable reference genes when working with intestinal tissue of sea bass larvae, regardless of their age and treatment. Accordingly, the genes ef1a and faua were found to be the most stable genes, followed by the genes *rp*s18 , rpl13a, *ywhab*, tubb2, gadph, actb′ and actb in their order of appearance for the combined samples. A pairwise variation analysis was performed to determine the most suitable number of reference genes for an accurate normalization (Fig. 3). Taking into account the cutoff value of 0.15 below which the inclusion of an additional reference gene is not required^14^, it was found that for all the groups, two reference genes are sufficient for accurate normalization.

NormFinder uses a model-based approach for identifying the optimal normalization gene among a set of candidates. In this model, estimations of both intra- and inter-group variation and a separate analysis of the sample subgroups in expression levels are included into the calculation of a gene expression stability value. NormFinder analysis was initially conducted on the different groups separately (Table 3). In the groups GF 10 dph and GF 16 dph *ef1a* and *faua* were identified as the most stable reference genes, whereas in the groups GFPr 10 dph and CON 16 dph *rps18* was identified as most stable reference gene followed by *ef1a* and *faua*, respectively.

Subsequently, the 40 samples were analyzed, while taking the groups into account in order to inspect the inter-group variation. Normfinder ranked *rpl13a* and *rps18* as the two best reference genes. However, according to the assumption behind the NormFinder algorithm, inclusion of potentially regulated candidate genes in the panel could interfere with the analysis. Therefore, all genes with a high inter-group variation (i.e. mean of gene intergroup variation >0.15) were disqualified, i.e. gadph, *actb*, *actb*′ and *tubb2*. Subsequently, analyses with Normfinder were repeated, taking only the genes with low inter-group variation into consideration, whilst ignoring the groups. The reference genes *faua* an *rpl13a* ranked best, followed by *rps18* and ef1a (Table 2). The variability obtained for the best pair of reference genes (faua/rpl13a), when the groups were taken into account, was 0.037 (Table 2).

## Discussion

In this study, an LCM protocol is described that successfully combines intestinal tract microdissection from fish larvae with downstream RNA analysis. Although LCM on mammalian tissue is a well-established technique in a large number of research fields, it is still emerging in aquaculture research^18^. Fish larvae, the weakest link in the aquaculture production cycle being primarily vulnerable to disease, are the emerging point of interest in nutrigenomics and pathogenomics^3^,^10^. However, their small size poses significant challenges to undertake genomic and transcriptomic studies. LCM of intestinal tissue, as pinpointed in the present study, will be a powerful new tool to study the molecular basis of host-pathogen interactions at the level of the intestine or the biochemical and physiological responses underpinning nutritional adaptations. As such, this tool may improve the efficiency and quality of fish larvae reared in a aquaculture setting. Furthermore, it can be deduced from previous mammalian studies that LCM is particularly indispensable to study the underpinning mechanisms of probiotics^11^,^19^. Although probiotics are believed to directly antagonize enteric pathogens, modulate innate or adaptive immunity, and strengthen mucosal barrier function, the specific molecules and pathways mediating these effects have yet to be identified. Basically, the LCM technique on fish intestinal tissue creates a myriad of opportunities to unravel the complex changes in gene expression that underlies nutritional and pathological remodeling of the intestinal tissue environment without interference of other organs or tissues.

LCM requires a combination of critical procedures including tissue collection, fixation, staining and microdissection, with each step having an impact on the subsequent RNA quality and morphological features^9^,^20^. It is widely accepted that extraction of intact RNA from paraffin-embedded tissue is a difficult procedure, often yielding low quality RNA^21^,^22^. However, methacarn does not contain aldehyde groups that may influence RNA integrity, making it the fixative of choice when paraffin embedment is required^22^,^23^,^24^. Cryopreservation on the other hand is the most suited method for molecular analysis when requiring the best possible RNA integrity. However, cryopreservation often decreases histomorphological quality, hampering a good identification of cell types in specific tissues^24^. When targeting intestinal tissue of sea bass, it was shown that cryopreservation is preferred, as the loss of histomorphological quality was limited compared to methacarn-fixation and the RNA quality was substantially higher.

The assessment of RNA quality is a critical step in obtaining reliable results from gene expression studies, as working with low quality RNA may seriously compromise the experimental results of downstream analysis^25^. Microfluidics-based electrophoresis systems, such as the Agilent 2100 bioanalyzer (Agilent Technologies, Santa Clara, California, USA) and the Experion automated electrophoresis system, that combine quantitation and quality assessment in a single step, are well established and widely used^26^. Both systems can accurately quantify and evaluate the integrity of samples with as little as 200 pg of total RNA. However, when working with lower amounts of RNA, which is common when using LCM, it is generally assumed that no reliable quality value can be provided^27^.

The 3′:5′ assay as proposed by Nolan & Bustin^28^ appeared to be particularly applicable for analysis of LCM samples when only a small amount of RNA is available. This assay starts with a reverse transcription based on oligodT primers only. When RNA is partially degraded, cDNA amplicons that are positioned further from the poly-A tail will be less abundant compared to amplicons near the poly A tail^29^. Therefore, a 3′:5′ ratio around 1 indicates high integrity, whereas a higher ratio suggests degradation. Nolan & Bustin^28^ set a cutoff ratio of 5 to suggest degradation. However, depending upon the assay, i.e. target sequence and primer pairs used, another threshold cutoff ratio of degraded RNA may be applicable. Therefore, in the current study, a degradation curve was constructed linking reliable RQI values with replicable 3′:5′ ratios. In this study, LCM samples with ratios lower than 2 were considered of good quality. The 3′:5′ assay was more sensitive compared to the Experion automated electrophoresis system, as all samples could be analyzed by means of the 3′:5′ assay, whereas most samples did not provide a reliable RQI value.

Gene expression measurement techniques such as quantitative reverse transcriptase RT-qPCR require a reliable normalization strategy to allow a meaningful comparison across biological samples, as is emphasized by the MIQE guidelines^15^. Erickson *et al*.^30^ evaluated three methods for normalizing gene expression in microdissection tissue samples, and concluded that reference genes are the most useful standard. So far, expression studies in the European sea bass have been performed with only one reference gene (either *actb*, *ef1a*, *rps18* or *gadph)*^4^,^5^,^31^,^32^. However, there is clear evidence that commonly used reference genes may significantly vary in expression over different experimental conditions, developmental stages and tissues^33^,^34^,^35^,^36^,^37^,^38^. As a consequence, it was suggested that at least two reference genes should be used for normalization and that these genes should be pre-validated using a larger set of reference genes (n≈10) to identify the most stable genes for each tissue/cell type and each experimental condition^16^. Recently, Mitter *et al*.^39^ published their findings on appropriate reference genes for expression studies in sea bass. Two reference genes *ef1a* and *rpl13a* were identified as suitable for expression analysis of eight developmental stages of sea bass. The reference genes *faua* and *rpl13a* were recommended as reference genes for expression analysis of a tissue panel (spleen, liver, kidney, and brain). Nevertheless, this study was performed using RNA samples obtained from whole sea bass body lysates. However, reference gene stability is cell type and tissue specific^14^,^16^. Therefore, homogeneous LCM tissue samples may offer an intuitively better foundation to identify reference genes suited for normalization of RT-qPCR directed on these specific tissues. In the current study we assessed the candidate reference genes in four experimental groups of larvae, of 10 LCM-collected samples each, and selected the best-suited reference genes from nine candidate genes. Expression stabilities of the reference genes were evaluated using geNorm and NormFinder. BestKeeper software was not considered as this program compares reference genes based on the raw Cq values, not taking the primer specific efficiencies into account, which may impact the ranking output of the reference genes^40^. With the GeNorm algorithm, the reference gene *faua* and *ef1a* were the most stable both within the groups as in the combined analysis, in accordance with Mitter *et al*.^39^. However, the ranking of Normfinder was more variable. The five least stable genes (*actb*, *gapdh*, *actb*′, *tubb2, ywhab*) were in all but one case ranked as least stable and in agreement with GeNorm, but the ranking of the more stable genes (*faua, rpl13a, rps18 and ef1A*) was more variable. The reason for this variable ranking may be their relatively small difference in stability measures compared to the other genes (Tables 2 and 3). Because of these small differences the relative impact of using a different set of these four genes will be minimal on the subsequent normalization.

It should be kept in mind that each new RT-qPCR experiment should be preceded by a careful validation of the reference genes using the larger set of genes described. Our results show that “classical” reference genes, i.e. actb, gadp and tubb2, are unsuitable for a correct normalization, as is in accordance with previous studies^41^,^42^. Notably, *rps18* was among the more stably expressed genes in our dataset. However, previous literature reported *rps18* as less suitable reference genes^39^,^43^, supporting the notion that these results cannot automatically be generalized to other tissues or experimental settings.

Although this protocol summarizes an LCM approach to study gene expression by RT-qPCR in intestinal tissue of sea bass larvae, it may be adapted to study larvae from a myriad of fish species or tissue types. LCM combined with transcriptomics implemented in aquatic nutritional, microbiological or immunological studies will contribute to render aquaculture more sustainable and economically appealing. However, it is important to take in consideration that some genes have a much lower expression, compared to reference genes. Therefore, with LCM reference gene Cq values approaching the SYBR green detection limits, the need to perform a linear amplification step may be inevitable, to ensure there is a detectable representation of even the weak-expressed transcripts. Alternatively, more sensitive techniques, such as digital PCR techniques may be used to minimize variation of the quantitative output of samples with low cDNA or DNA levels^44^,^45^.

In conclusion, we demonstrate that a careful optimization of the LCM procedure allows recovery of high quality mRNA from complex intestinal tissues. In the current study *ef1a*, *faua, rpl13a* and *rps18* were the most stably expressed genes and as such most suitable to be implemented as reference genes for an appropriate normalization of RT-qPCR data obtained from intestinal tissue of sea bass larvae.

## Materials and Methods

During all processes, precautions were taken to minimize the risk of RNase contamination. All working areas were treated with RNase inhibitors (RNase AWAY® Reagent, Ambion®). Glassware was heated at 180 °C overnight. Certified RNase free disposables were used during each step. Diethyl pyrocarbonate (DEPC) water was prepared by mixing 0.1% Diethyl pyrocarbonate (Sigma Aldrich, Diegem, Belgium) with ultrapure water, followed by incubating overnight at room temperature and autoclaving. All experiments were performed in compliance with the MIQE guidelines^17^. All experiments were approved by the Ethical Committee of the Faculty of Veterinary Medicine and Bioscience-Engineering, Ghent University (no. EC2013/19) and carried out in accordance with the approved guidelines and legislation in force.

### Larval specimens

Sea bass eggs were obtained from the Ecloserie Marine de Gravelines (Gravelines, France). Three groups of larvae were raised: conventional larvae (CON), germ-free larvae (GF) and germ-free larvae supplemented with a probiotic candidate (GFPr). For culturing the CON larvae, the eggs were placed into 500 ml glass incubation bottles containing 400 ml of autoclaved artificial seawater (AASW), adjusted to a salinity of 33 ppt and a temperature of 16 ± 1 °C. A low level of filtered (0.2 μm, Sartorius AG, Göttingen, Germany) sterile air was provided to all incubation bottles. Hatched larvae were transferred to 24-well plates, one larva per well containing 2 ml of filtered AASW, of which one ml was changed every other day. Starting at 7 dph until sampling, larvae were fed live sterile *Artemia franciscana* (Linnaeus, 1758) nauplii (20–30 per well) every other day, originating from the Great Salt Lake, Utah (INVE Aquaculture NV, Dendermonde, Belgium). The larvae underwent a circadian rhythm of 8 hours light and 16 hours darkness. To obtain GF larvae, the eggs were subjected to two successive rounds of three minutes submersion in a 400 ppm glutaraldehyde solution (50 wt% solution in water, Merck KGaA, Darmstadt, Germany) in AASW and subsequently collected and placed into 500 ml glass incubation bottles containing 400 ml of AASW supplemented with a mix of antimicrobial agents^46^. Subsequently, the larvae were transferred to 24-well plates and maintained further as described for the CON larvae, except that the GF larvae were housed in a barrier isolator with a glove system (G(ISO)-T3, TCPS, Rotselaar, Belgium). The GFPr larvae were reared as the GF larvae, except for the additional administration of *Vibrio lentus*, a probiotic candidate strain (unpublished data) at 4, 6 and 8 dph at 10^7 colony forming units*ml^−1^. All larvae were euthanized with an overdose of MS-222 (Sigma-Aldrich, Diegem, Belgium) and fixed immediately as described below.

### Optimization of the fixation protocol

Snap freezing was compared to chemical fixation with methacarn in terms of preserving morphological features and RNA integrity.

#### Fixing, processing and staining

For snap freezing, ten CON larvae of 16 dph were embedded in their entirety in a gelatin capsule (Capsules gelatin, Electron Microscopy Sciences, Hatfield, USA) filled with cryopreservative solution (Tissue Tek® O.C.T^TM^ Compound; Sakura, Alphen aan den Rijn, The Netherlands), snap frozen in liquid nitrogen and stored at −80 °C. The basic protocol outlined by Espina *et al*. (2006) was used, but modified as detailed below. Before sectioning, the cryostat (Leica sectioning crysostat) was wiped down with 100% ethanol to avoid cross contamination, and a fresh disposable blade was used to cut each sample. The tissue was placed in the cryostat for about 10 min to adjust to the cutting temperature (−25 °C to −30 °C). Transverse tissue sections (7 μm) of the complete larvae were cut and mounted on Histobond® Adhesion Microscope Slides (Marienfeld, Lauda-Königshofen, Germany). The slides were stored in the cryostat at −25 °C until the cutting was completed. Slides were fixed immediately in RNase free 70% ethanol (10 s) and rehydrated in DEPC treated water (10 s). The tissue slides were then stained in haematoxylin (Merck KGaA, Darmstadt, Germany) for 10 s, followed by rinsing in DEPC treated water (10 s) and Scott’s tap water solution (10 s) (Sigma-Aldrich, Diegem, Belgium). Subsequently, the tissue slides were stained with eosin Y (Merck KGaA, Darmstadt, Germany) for 5 s and dehydrated in graded ethanol concentrations, starting with two baths of 95% for 10 s followed by two baths of isopropanol for 30 s. Finally, the sections were completely dehydrated by immersing the slides in two successive baths of xylene for 60 s each. The slides were air dried in a laminar flow cabinet and individually placed in sterile 50 ml falcon tubes to prevent rehydration by air.

For methacarn fixation, the whole body of ten CON larvae of 16 dph were fixed in modified methacarn (methanol: glacial acetic acid = 8:1) for 4 hours and routinely processed to paraffin embedding with an automatic processor (Microm STP 420D, Thermo Fisher Scientific, Massachusetts, USA). Paraffin-embedded tissues were cut into 8 μm thick sections and stored for a maximum period of two days at 4 °C. Slides were dewaxed, rehydrated, and then stained with haematoxylin and eosin using the protocol described above. To remove the paraffin wax, slides were immersed in two consecutive baths of xylene for 1 min each. After staining, slides were rapidly air dried in a laminar flow cabinet and placed individually in 50 ml falcon tubes.

#### Assessment of morphological features

The morphological features of the obtained tissue sections were assessed blindly by two histologists applying a scale 0 to 3 (0 = very poor; 1 = poor; 2 = fair and 3 = good). The scores were based on the following criteria with focus on the intestinal tissue: intact cellular and nuclear morphology, clearly visible brush border, distinct basal lamina and staining quality. In addition, the overall morphology, i.e. the morphology of all tissues in the tissue section, was evaluated.

#### Evaluation of RNA integrity

RNA was prepared by using a sterile scalpel blade to gently scrape the tissue off the tissue slide. Tissue was captured with a 5 μl glass micropipette and transferred into 50 μl of extraction buffer, in a DNA Lo-Bind MicrocentrifugeTube (Eppendorf, Hamburg, Germany)^47^. A new scalpel blade was used for each sample. RNA was extracted using the PicoPure RNA Isolation Kit (Arcturus engineering, Mountain View, California, USA) according to the manufacturer’s protocol, and included an on-column RNase-free DNAse treatment (Qiagen, Venlo, The Netherlands). The column was washed and total RNA was eluted in 20 μl of elution buffer. Isolated RNA was frozen at −80 °C until further processing. Total RNA integrity was assessed using the Experion™ Automated Electrophoresis System with the Experion RNA HighSens analysis kit (Bio-Rad, Hercules, California, USA).

### Laser capture micro-dissection

The fixation protocol based on snap freezing gave the best results regarding to maintenance of adequate morphology and RNA integrity and was therefore adopted in the further continuation of the LCM protocol development.

The Pixcell IIe IR laser capture (Arcturus engineering, Mountain View, California, USA) was used to microdissect intestinal tissue from 10 CON larvae at 16 dph, ten GF larvae at 10 dph, 10 GF larvae at 16 dph and ten GFPr larvae at 10 dph. Laser settings were chosen to maximize the size of the laser spot without contaminating the sample with non-target tissue. A laser spot size of 30 μm diameter was selected, with 20 mW in power and 5 ms pulse duration. Efficiency of capturing was evaluated by examining excised cell fragments on the CapSure*®* Macro LCM Caps (Arcturus engineering, Mountain View, California, USA) and the tissue on the slide before and after lifting off the cap. Debris or excess section material was removed to the maximum possible extent by smoothly touching the film with a post-it strip. After tissue collection, the cap was put on a Lo-Bind Microcentrifuge Tube and incubated in 50 μl of RNA extraction buffer. Tubes were kept with the cap down, until all tissue was collected. The whole procedure, from staining to finishing LCM, did not exceed 30 min.

### RNA integrity assessment of LCM samples

Total RNA was extracted by using the PicoPure RNA Isolation Kit according to the manufacturer’s instructions. Genomic DNA was eliminated by an additional on-column RNase-free DNase treatment. The column was washed and total RNA was eluted in 20 μl of elution buffer. Isolated RNA was frozen at −80 °C until further processing. Total RNA integrity and quantity were assessed using the Experion™ Automated Electrophoresis System with the Experion RNA HighSens analysis kit. When the RNA concentration was too low to accurately determine an RQI value, the cutoff value was overruled by adapting the program settings and unreliable RQI values were displayed between brackets. Subsequently, RNA integrity was assessed by the alternative, PCR-based 3′:5′assay. For cDNA synthesis, 2 μl of total RNA was reverse transcribed in a final volume of 20 μl using goscript^TM^ Reverse Transcription System (Promega, Leiden, The Netherlands) in a mix containing 1 unit/μl reverse transcriptase, 4μl RT buffer, 2.5 mM MgCl_2_, 0.1 mM dNTP’s and 0.1 μg/μl oligodT’s. Reverse transcriptase was performed during 60 min at 42 °C. Finally, to remove PCR inhibitors, cDNA was purified using the genelute^TM^ PCR clean-up kit (Sigma-Aldrich, Diegem, Belgium)^48^. Two primer pairs were designed for *actb* using Primer3 software. One primer set was designed to amplify the region near the 5′end of the RNA and the second set was designed more towards the 3′end (Table 4). The efficiency of each primer pair was calculated according to the standard curve method using the equation E = 10^(−1/slope)^ − 1 and the specificity of the PCR products was assessed by melting-curve analysis. Quantitative PCR was performed using the Bio-Rad CFX96 real-time system (Bio-Rad, Hercules, California, USA) with the SsoAdvanced SYBR Green master mix (Bio-Rad, Hercules, California, USA) according to the manufacturer’s instructions for 20-μl samples containing 2 μl of undiluted cDNA. The concentration of primer pairs used is depicted in Table 4. Thermocycling conditions were as follows: 98 °C for 30 s followed by 40 cycles of 95 °C for 30 s, 60 °C for 30 s, 72 °C for 30 s and finally a melting curve analysis was performed beginning at 65 °C for 5 s with a gradual increase in temperature (0.5 °C/5 s) to 95 °C. No-template controls (NTC) were included in all qPCR runs and did not record any positive Cq values. Standard curves were generated for each primer pair with serial 5-fold dilutions of cDNA prepared using whole body CON 16 dph sea bass lysate RNA, mixed with pooled RNA from the 40 LCM samples of intestine of sea bass larvae. Data were analyzed using the CFX manager^TM^ software 3.1 (Bio-Rad, Hercules, California, USA). All samples were amplified in duplicate and the mean was implemented within further calculations.

The ratio of relative quantity of both amplicons of the *actb* mRNA reflects the RNA integrity of the transcript^28^. Consequently, a 3′:5′ratio of around 1 indicates high integrity. The ratio is calculated as follows: E^ − ΔCq(3′)/ E^ − ΔCq(5′) with ΔCT = Ct(sample)-Ct(standard curve). To determine the ratio corresponding with acceptable quality, RNA samples from whole body CON 16 dph sea bass were diluted, sequentially degraded and assessed by using the Experion system. Degradation was induced by exposure to 80 °C and every 10 minutes a sample was processed and this for nine time points.

### Reference gene evaluation

Nine candidate reference genes were picked for data normalization according to Mitter *et al*.^39^ [glyceraldehyde-phosphate-dehydrogenase (*gapdh*), β-actin (*actb and actb*′), 40S ribosomal protein S30 (*faua*), ribosomal protein l13a (*rpl13a*), β2-tubulin (*tubb2*), tyrosine 3 monooxygenase/tryptophan 5-monooxygenase activation protein (*ywhab*), ribosomal Protein S18 (*rps18*) and translation elongation factor (*ef1a*)] (Table 4). For cDNA synthesis, 5 μl of total RNA from each of the 40 samples was reverse transcribed in a final volume of 5  μl using qScript™ cDNA Supermix (Quanta BioSciences, Gaithersburg, USA) according to the manufacturer’s recommendations. Reverse transcriptase was performed during 30 min at 42 °C. To remove PCR inhibitors cDNA was purified using the genElute^TM^ PCR clean-up kit^48^. Quantitative PCR was performed using the CFX96 Real-Time PCR detection system. Three SYBR Green master mixes were tested under the conditions as described by Mitter *et al*.^39^. Under these conditions the SsoAdvanced^TM^ SYBR® Green Supermix gave the best sensitivity and efficiency, with no need to further optimize the PCR conditions. SsoAdvanced^TM^ SYBR® Green Supermix was used according to the manufacturer’s instructions for 20 μl samples containing 2 μl of undiluted cDNA. The primer pair concentrations are depicted in Table 4. Thermocycling conditions were as follows: 98 °C for 30 s followed by 40 cycles of 95 °C for 30 s, 57 °C for 60 s. Finally, a melting curve analysis was performed beginning at 65 °C for 5 s with a gradual increase in temperature (0.5 °C/5 s) to 95 °C. NTC’s were included in all qPCR runs and no positive Cq values were recorded. Standard curves were generated for each primer pair with serial

5-fold dilutions of cDNA prepared using whole body CON 16 dph sea bass lysate RNA, mixed with pooled RNA from the 40 LCM samples of intestine of sea bass larvae. For each primer the PCR efficiency was calculated according to the standard curve method using the equation E = 10^[−1/slope]^ − 1) × 100 and the specificity of the PCR products was controlled by melting-curve analysis (Table 4). Data was analyzed using the CFX manager^TM^ software 3.1 (Bio-Rad, Hercules, California, USA). All samples were amplified in duplicate and the mean was implemented within further calculations.

GeNorm^14^ and Normfinder^49^ were used to evaluate the expression stability of each candidate reference gene. For both tools raw Cq values were transformed into relative quantification data using the ΔCq method. Briefly, the highest Cq value for each gene was set to 1 and values for the rest of the samples were calculated relative to this value. Following, for each data point, the equation E^(−ΔCq) was applied. These expression quantities were the input data for geNorm and Normfinder.

## Additional Information

**How to cite this article**: Schaeck, M. *et al*. Laser capture microdissection of intestinal tissue from sea bass larvae using an optimized RNA integrity assay and validated reference genes. *Sci. Rep*. **6**, 21092; doi: 10.1038/srep21092 (2016).

## Figures and Tables

**Table 1:** 3′:5′ratio calculation, RQI values and RNA concentrations of LCM samples of sea bass larval intestinal tissue.

Groups	ΔCq 3′	ΔCq 5′	3′:5′ Ratio*	RQI	Conc. (pg)
GF 10 dph	7.7	8.6	2.0	[8.4]	178
GF 10 dph	8.5	8.5	1.0	[4.9]	108
GF 10 dph	7.2	7.9	1.7	[8.3]	159
GF 10 dph	8.6	8.8	1.1	[7.1]	159
GF 10 dph	6.7	5.9	0.6	7.1	378
GF 10 dph	6.9	6.7	0.9	7.7	334
GF 10 dph	8.2	9.2	2.2	[5.9]	100
GF 10 dph	8.3	9.4	2.3	7.1	303
GF 10 dph	5.8	6.6	1.8	7.3	410
GF 10 dph	6.3	7.5	2.4	[8.9]	197
GFPr 10 dph	9.1	9.3	1.3	[5.4]	69
GFPr 10 dph	7.9	8.6	1.7	[3.3]	102
GFPr 10 dph	6.7	7.2	1.4	[10]	65
GFPr 10 dph	6.7	7.4	1.7	[8.2]	57
GFPr 10 dph	8.2	9.1	2.0	[7.5]	135
GFPr 10 dph	7.9	8.4	1.5	[5]	112
GFPr 10 dph	7.2	7.8	1.6	[9.4]	171
GFPr 10 dph	7.4	7.4	1.1	[7.8]	62
GFPr 10 dph	6.3	6.4	1.1	[8.1]	207
GFPr 10 dph	8.1	7.9	0.9	7.2	284
CON 16 dph	8.0	8.4	1.4	[5.5]	139
CON 16 dph	7.4	7.1	0.8	[6.5]	164
CON 16 dph	8.0	7.8	0.9	[6.8]	128
CON 16 dph	9.2	9.4	1.2	[4.3]	121
CON 16 dph	7.0	7.0	1.0	[9.1]	170
CON 16 dph	8.6	8.4	0.9	[8.8]	100
CON 16 dph	8.5	8.4	0.9	[5.7]	114
CON 16 dph	7.9	7.8	0.9	[5.9]	125
CON 16 dph	8.9	8.6	0.8	[7.8]	95
CON 16 dph	9.1	8.8	0.8	[5.3]	105
GF 16 dph	6.8	6.0	0.6	[5.2]	167
GF 16 dph	8.0	7.4	0.7	[6.5]	139
GF 16 dph	8.0	7.0	0.5	[5.8]	105
GF 16 dph	8.8	8.4	0.8	[5.3]	144
GF 16 dph	8.1	7.5	0.7	[7.5]	105
GF 16 dph	7.1	6.9	0.9	[6.2]	168
GF 16 dph	9.1	8.4	0.6	[5.9]	112
GF 16 dph	8.8	8.3	0.7	[4.9]	106
GF 16 dph	8.5	7.6	0.5	8.9	531
GF 16 dph	5.3	5.4	1.1	8.4	421

Unreliable RQI values are depicted in brackets.

*3′:5′ Ratio = E3′^(−ΔCq 3′) / E5′^(−ΔCq 5′).

ΔCq 3′ = mean Cq 3′ samplen - mean Cq 3′ standard curve.

ΔCq 5′ = mean Cq 5′ samplen - mean Cq 5′ standard curve.

CON: conventional larvae; GF: germ-free larvae and GFPr: germ-free larvae supplemented with a probiotic candidate.

**Table 2:**
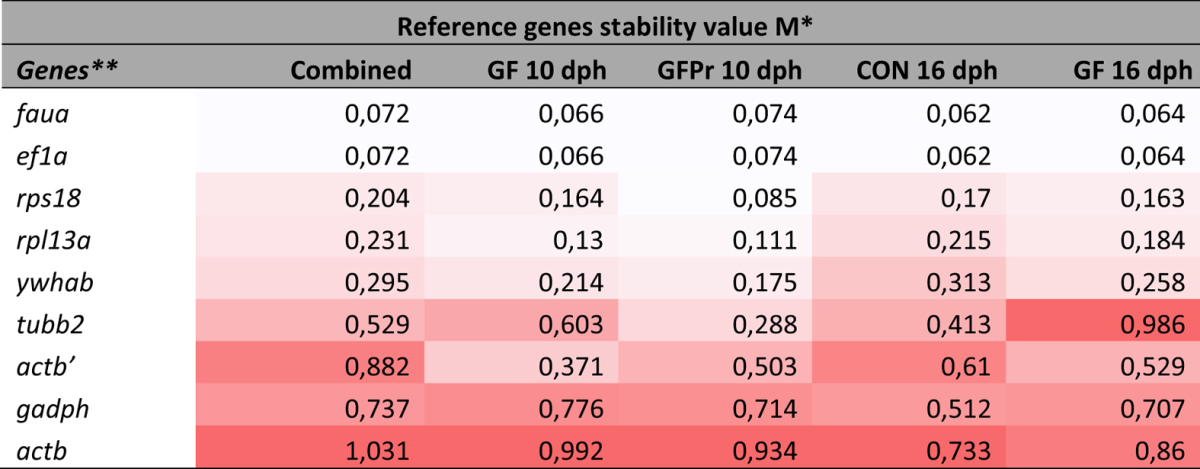
Ranking of the candidate reference genes according to their stability value using geNorm.

*M increases from white (lowest group value) to red (highest group value).

**Reference genes are ranked according to the mean M.

CON: conventional larvae; GF: germ-free larvae and GFPr: germ-free larvae supplemented with a probiotic candidate.

**Table 3:**
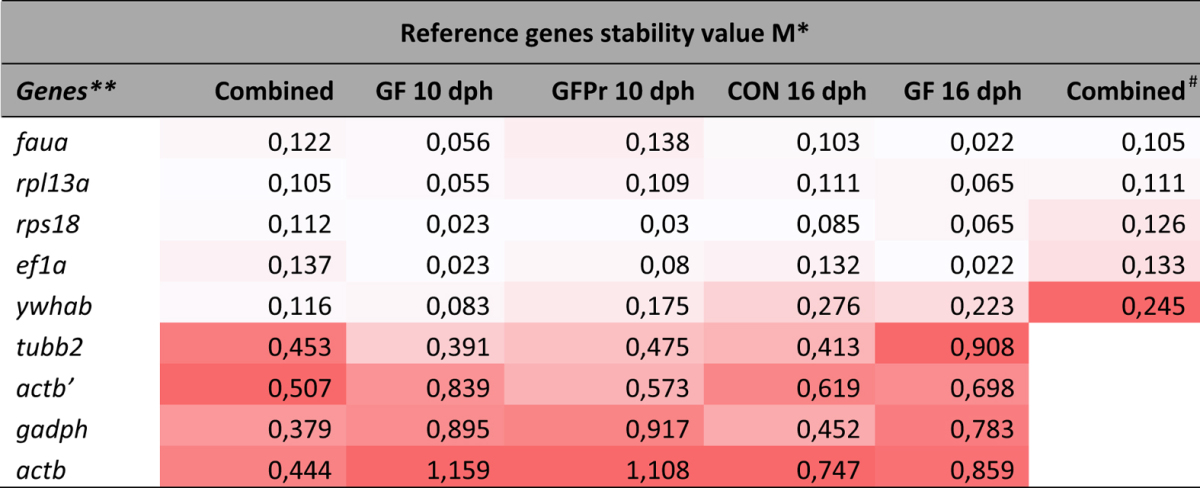
Ranking of the candidate reference genes according to their stability value using NormFinder.

*M increases from white (lowest group value) to red (highest group value).

**Reference genes are ranked according to the mean M.

^#^All genes with a high inter-group variation (cf. combined) were disqualified (i.e. *gadph, actb, actb*′ and *tubb2)* and analyses were repeated.

CON: conventional larvae; GF: germ-free larvae and GFPr: germ-free larvae supplemented with a probiotic candidate.

**Table 4:** Gene name, primer name, accession number, primer sequence, amplicon length, used primer concentration, annealing temperature and encountered efficiency of each candidate reference gene and the 3′:5′ assay target sequence used in the present study.

Gene name	Primer name	Accession nr.	Primer sequence	Amplicon length	Primer conc. (μM)	Ta (°C)	Efficiency (%)
*actb*	*actb*	AJ537421.1	F: CTGGGATGACATGGAGAAGA R: CTTGATGTCACGCACGATTT	406	0.32	57	100
*actb*	*actb*′	AJ537421.1	F: GTGCGTGACATCAAGGAGAA R: GCTGGAAGGTGGACAGAGAG	436	0.04	57	88
*faua*	*faua*	FM004681	F: GACACCCAAGGTTGACAAGCAG R: GGCATTGAAGCACTTAGGAGTTG	149	0.03	57	101
*rpl13a*	*rpl13a*	DT044539	F: TCTGGAGGACTGTCAGGGGCATGC R: AGACGCACAATCTTGAGAGCAG	148	0.08	57	100
*tubb2*	*tubb2*	FM003484	F: GCCTCAGGTGGCAAATATGT R: CCTCAGTGTAGTGACCCTTG	166	0.32	57	106
*ywhab*	*ywhab*	AM973424	F: TCTGCCAGGACGTGCTGAACC R: TCCACCGTATCCTTCTTTGAGTC	151	0.16	57	97
*ef1a*	*ef1a*	FM019753	F: AAATGCGGAGGAATCGACAA R: GAGCCCTTGCCCATCTCAG	70	0.32	57	102
*rps18*	*rps18*	AM490061	F: AGGGTGTCGGCAGACGTTAC R: CTTCTGCCTGTTGAGGAACC	163	0.16	57	98
*gapdh*	*gapdh*	AY863148	F: GTGCCAGCCAGAACATCAT R: TGTCGTCATATTTGGCGGGTTTC	171	0.32	57	100
*actb*	*actb*5′	AJ537421.1	F: CTGAACTACCCCATCGAGCA R: *GACTTGGGGTTTCGGTTGTC*	149	0.2	60	100
*actb*	*actb3*′	AJ537421.1	F: *AAGATCATTGCCCCACCTGA* R: *CCTGCTTGCTGATCCACATC*	100	0.2	60	99

Note that the actb primers used for the normalization (act and actb′) are different from the actb primers used for the 3′:5′ assay (actb5′ and actb3′).
